# Monoamine Oxidase-Related Vascular Oxidative Stress in Diseases Associated with Inflammatory Burden

**DOI:** 10.1155/2019/8954201

**Published:** 2019-04-15

**Authors:** Adrian Sturza, Călin M. Popoiu, Mihaela Ionică, Oana M. Duicu, Sorin Olariu, Danina M. Muntean, Eugen S. Boia

**Affiliations:** ^1^Department of Functional Sciences–Pathophysiology, Faculty of Medicine, “Victor Babeș” University of Medicine and Pharmacy, Timișoara, Romania; ^2^Center for Translational Research and Systems Medicine, Faculty of Medicine, “Victor Babeș” University of Medicine and Pharmacy, Timișoara, Romania; ^3^Department of Pediatric Surgery, Faculty of Medicine, “Victor Babeș” University of Medicine and Pharmacy, Timișoara, Romania; ^4^Department of Surgery I, Faculty of Medicine, “Victor Babeș” University of Medicine and Pharmacy, Timișoara, Romania

## Abstract

Monoamine oxidases (MAO) with 2 isoforms, A and B, located at the outer mitochondrial membrane are flavoenzyme membranes with a major role in the metabolism of monoaminergic neurotransmitters and biogenic amines in the central nervous system and peripheral tissues, respectively. In the process of oxidative deamination, aldehydes, hydrogen peroxide, and ammonia are constantly generated as potential deleterious by-products. While being systematically studied for decades as sources of reactive oxygen species in brain diseases, compelling evidence nowadays supports the role of MAO-related oxidative stress in cardiovascular and metabolic pathologies. Indeed, oxidative stress and chronic inflammation are the most common pathomechanisms of the main noncommunicable diseases of our century. MAO inhibition with the new generation of reversible and selective drugs has recently emerged as a pharmacological strategy aimed at mitigating both processes. The aim of this minireview is to summarize available information regarding the contribution of MAO to the vascular oxidative stress and endothelial dysfunction in hypertension, metabolic disorders, and chronic kidney disease, all conditions associated with increased inflammatory burden.

## 1. Introduction

Oxidative stress and low-grade inflammation are widely recognized as inextricably linked pathomechanisms of chronic cardiometabolic and kidney diseases that evolve as rampant pandemics of our century. Oxidative stress is currently viewed not merely as an imbalance of prooxidants/antioxidants but also as a perturbation of redox signaling and control [1]. Redox signaling consists in reversible changes associated with the generation of low amounts of reactive oxygen species (ROS) responsible for the activation of intracellular signaling pathways, whereas oxidative stress is always related to high levels of ROS production that causes irreversible tissue injury. The occurrence of complex networks of redox signaling and amplification loops of ROS generation in pathology, or the so-called phenomenon of “ROS-induced ROS release,” is responsible for the difficulty of studying redox pathophysiology in clinical settings [2]. Since both redox signaling and oxidative stress lead to the release of proinflammatory mediators, the process of “ROS-induced inflammation” has lately emerged as a novel contributor to the progression of the vast majority of chronic diseases [3]. Inflammation, in turn, is responsible for a prooxidative status via ROS generation by the activated monocytes/macrophages, that is, the activation of inflammasomes [4] with subsequent induction of a self-perpetuating, vicious circle termed “ROS-induced and inflammation-induced oxidative stress” leading to cell injury and death [3].

ROS are generated in most of the cells from both enzymatic and nonenzymatic sources. While NADPH oxidases, uncoupled eNOS, xanthine oxidase, mitochondrial respiratory chain, lipooxygenase, cyclooxygenase, and myeloperoxidase are widely recognized as the classical sources of vascular ROS [5–7], research carried out in the past 2 decades highlighted the role of monoamine oxidases (MAO) as important sources of oxidative stress in the heart [8–10] and in vessels [11–13].

**Figure 1:**
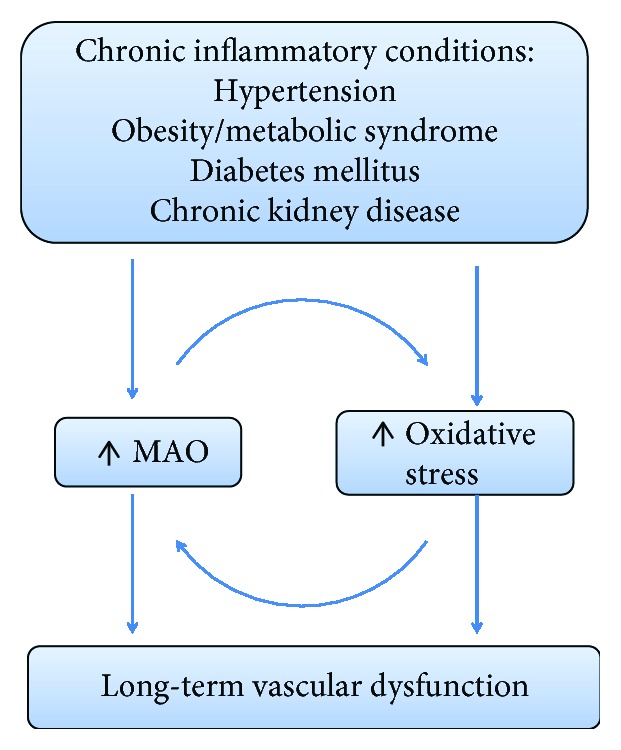
MAO is a mediator of endothelial dysfunction in conditions associated with increased inflammatory burden (hypertension, obesity, diabetes, and chronic kidney disease).

The aim of this minireview is to summarize the main results regarding the contribution of MAO to the vascular oxidative stress and endothelial dysfunction in chronic diseases associated with increased inflammatory burden (Figure 1) and to highlight the role of MAO inhibitors as potential candidates in drug repurposing.

## 2. MAO: Distribution and Function

### 2.1. MAO Tissue Distribution

Monoamine oxidase (MAO) was discovered back in 1928 by Hare [14] in the rabbit liver where it catalyzed the oxidation of tyramine (hence, it was named tyramine oxidase at first), and it was reported to be located at the outer mitochondrial membrane by Schnaitman et al. [15] in 1967. Later, genetic studies unequivocally demonstrated the existence of two individual membrane-bound flavoproteins (that use FAD as a cofactor), MAO-A and MAO-B, encoded by 2 distinct genes with 70% identity in humans [16].

MAO have variable, species-dependent expression in several tissues and organs, e.g., heart, vasculature, liver, intestine, lung, kidney, thyroid gland, platelets, and placenta [17, 18]; most of them contain both isoforms with two exceptions: MAO A is solely expressed in placental mitochondria, while MAO-B is found only in platelets [19].

### 2.2. Roles of MAO in the Nervous System

The physiological role of MAO is related to the metabolism of endogenous monoaminergic neurotransmitters and exogenous biogenic amines in the central nervous system and peripheral tissues, respectively. Accordingly, catecholamines (norepinephrine and epinephrine), serotonin, and melatonin are preferentially metabolized by MAO-A, while phenylethylamine and benzylamine are largely oxidized by MAO-B. Both isoenzymes catalyze the deamination of tyramine, dopamine, octopamine, and tryptamine [19].

The oxidative deamination of a monoamine by mitochondrial MAO generates (i) a corresponding aldehyde as the primary product, which is rapidly metabolized to a carboxylic acid and excreted to prevent toxicity; (ii) hydrogen peroxide, resulting from the catalytic FAD-FADH_2_ cycle that will be inactivated by catalase in the peripheral tissues and glutathione peroxidase in the brain; and (iii) ammonia, as the third potential toxic by-product [20].

In the central and peripheral nervous systems, intraneuronal MAO-A and B protect neurons from exogenous amines, prevent the actions of endogenous neurotransmitters, and regulate the intracellular amine content. In peripheral tissues, MAO is involved in the oxidative catabolism of amines and prevent the penetration of dietary amines (such as tyramine from cheese and fermented drinks) into the circulation [21].

The vast majority of research in the field focused on the role of MAO in the nervous system. In line with their role in the inactivation of neurotransmitters, abnormal expression of MAO is considered to be responsible for a couple of psychiatric and neurological disorders and the treatment with MAO inhibitors has been available for more than 50 years in neuropsychiatric disorders [21–23]. Thus, selective inhibitors of MAO-B (selegiline, rasagiline, and safinamide) are indicated in the treatment of Parkinson disease, whereas selective MAO-A inhibitors (moclobemide) act as antidepressants. Lately, the reversible (selective) inhibitors are increasingly used since they are devoid of the side effects of the irreversible MAO inhibitors [24].

### 2.3. Roles of MAO in the Peripheral Tissues

The identification of MAO-A and B in the peripheral tissues prompted the research on their role in regulating bioamine inactivation, in particular, of serotonin (5-hydroxytryptamine) and norepinephrine, in the cardiovascular system (reviewed in refs. [8, 10, 25]).

Early studies brought 2 major insights to the field. Firstly, MAO activity was found to be associated with both neuronal and extraneuronal compartments in the mammalian heart; indeed, in humans MAO was responsible for norepinephrine degradation both in the cardiac sympathetic nerve endings and (mainly through MAO-A) in cardiomyocytes [26, 27]. Second, MAO expression/activity was reported to be compensatingly increased in animal models, which was associated with increased activation of the sympathetic nervous system and high substrate (norepinephrine) availability, such as in hypertension [28], diabetes [29], and ageing [30].

The more recent, ongoing studies tackled the role of MAO as a constant source of increased H_2_O_2_ generation and subsequent oxidative stress in both experimental models of disease [10, 31, 32] and humans with cardiovascular pathology [33–35]. We and others have systematically addressed the contribution of MAO to endothelial dysfunction in age-related pathologies and the beneficial effects of MAO inhibitors (MAOI) in alleviating it [12, 13, 33, 36–38]. Accordingly, the role of MAO-related oxidative stress in the vascular system in hypertension, obesity/metabolic syndrome, diabetes, and chronic kidney disease will be discussed. We will also present preliminary data relevant for the role of inflammation in increasing the vascular expression of MAO-A.

## 3. MAO and Inflammation

The role of vascular inflammation as part of the pathophysiology of cardiovascular disease is widely acknowledged as being intimately linked with oxidative stress, endothelial dysfunction, and the progression of atherosclerosis [39, 40].

Currently, the MAO-inflammation connection is far from being elucidated. In an elegant study aimed at providing mechanistic insights into the pathomechanisms of diabetic cardiomyopathy and inflammation, Deshwa et al. showed that coexposure of cardiomyocytes to high glucose and a proinflammatory cytokine (IL-1) elicited MAO-related oxidative stress with subsequent mitochondrial dysfunction and endoplasmic reticulum (ER) stress [41].

As for the role of vascular MAO, it has already been reported that ex vivo stimulation and *in vivo* treatment with lipopolysaccharide (LPS) resulted in the upregulation of both MAO isoforms in mice [13] and rat [42] aortic rings via a signal transduction pathway that appears to involve NF*κ*B and PI3 kinase. In these vascular samples, MAO inhibition was able to partially normalize the vasomotor function and decrease ROS generation [13]. Interestingly, MAO upregulation triggered by LPS was mentioned in the context of a rat periodontal disease model and treatment with the MAO inhibitor, phenelzine, was able to significantly reduce the amount of H_2_O_2_ [43]. Another recent study showed that moclobemide, a reversible MAO-A inhibitor, was able to attenuate vascular inflammation of intramyocardial arteries in a rat model of ischemia-reperfusion injury [44]. Interestingly, it has been reported that MAO-A is involved in ROS generation in alternatively activated monocytes/macrophages [45, 46].

**Figure 2:**
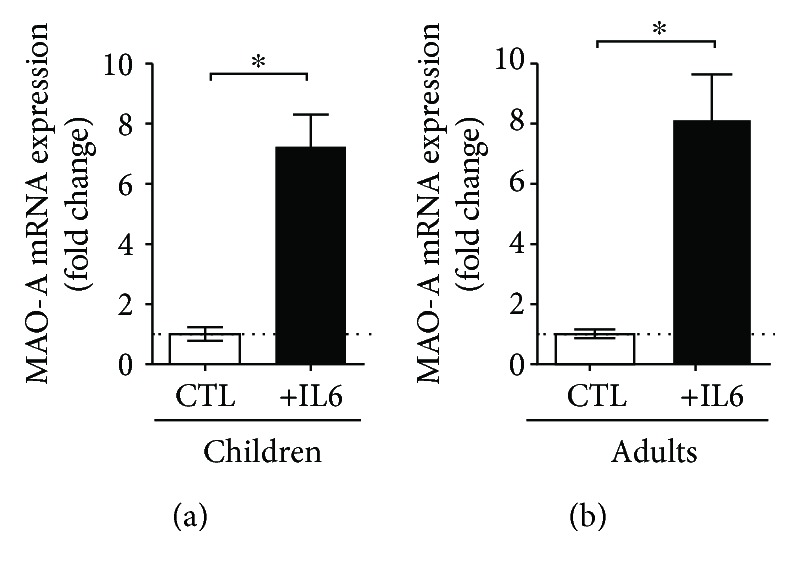
Stimulation with IL-6 (100 ng/ml, 12 h) increases MAO-A expression in mesenteric artery branches harvested from patients undergoing elective abdominal surgery (mRNA level was examined by real-time RT-PCR, *n* = 6, ^∗^*p* < 0.05 CTL vs. IL6).

In keeping with the translational approach, we have recently noticed that using IL-6 (100 ng/ml, 12 h) to stimulate mesenteric artery branches isolated from patients (both children and adults) subjected to abdominal surgery led to increased MAO expression (Figure 2).

## 4. MAO and Hypertension

Hypertension is a multifactorial disease involving the increase in peripheral vascular resistance and/or cardiac output as direct consequences of the renin-angiotensin-aldosterone system upregulation and sympathetic system activation with the early occurrence of endothelial dysfunction [47].

In the setting of increased oxidative stress, the main mechanism responsible for endothelial injury and progression of cardiometabolic diseases is represented by the impairment of the NO signaling cascade [48]. In this process, the role of angiotensin II- (Ang II-) induced endothelial dysfunction and cardiovascular remodeling/fibrosis has been systematically documented over the past decades [49, 50]. Indeed, Ang II is a potent vasoconstrictor, and when overexpressed, it also promotes inflammation and vessel damage via stimulating ROS production, with all the deleterious effects being mediated by the AT_1_ receptor [51]. In particular, ROS generation through the activation of vascular NAD(P)H oxidases (Nox) has been documented [52].

Ang II also augments the production of mitochondrial ROS, but the intimate molecular mechanisms are far from being elucidated [49]. Increased expression of Ang II in the brain has also been reported to stimulate ROS generation by NADPH oxidase, mitochondrial electron transport chain, and proinflammatory cytokines which ultimately leads to an increase in neuronal activity and sympathetic outflow, respectively [53].

Less is known about the effect of Ang II on MAO. Thus, the MAO-Ang II interaction has been reported earlier to occur in the central nervous system. In a pioneering study on hypothalamus and brain stem cell cultures, Sumners et al. reported that Ang II increased MAO activity and neuronal norepinephrine uptake, an effect mediated by Ang II receptors [54]. Moreover, when spontaneously hypertensive rats were subjected to chronic treatment with ACE inhibitors (captopril and enalapril) or the AT-1 receptor blocker (candesartan), MAO activity in the heart was decreased, whereas noradrenaline and adrenaline contents doubled in the left ventricle; interestingly, the effects of Ang II and the pharmacological inhibitors on MAO activity on cardiac tissue *in vitro* could be recapitulated [55].

In an earlier study addressing the contribution of MAO to vascular oxidative stress in mice, an upregulation of both MAO-A and B expression in aortic rings after both *in vivo* stimulation with Ang II (by minipumps) and ex vivo treatment was reported. In these vascular samples, incubation with MAO-A and B inhibitors was able to improve the vasomotor function and decrease H_2_O_2_ generation, respectively [13].

The Ang II-dependent increase in MAO-A activity that was reported to occur in HL-1 cardiomyocytes acutely exposed (18 h) to submicromolar concentrations of Ang II could be prevented in the presence of the AT-1 receptor antagonist, irbesartan. An increased activity of MAO-A (and also of the catabolic enzymes, catalase and aldehyde dehydrogenase) was reported by the same group in left ventricular cardiomyocytes isolated from streptozotocin-treated rats after 2 weeks of diabetes. Interestingly, when the animals were chronically treated with the AT-1 receptor antagonist losartan, activation of MAO-A and aldehyde dehydrogenase (but not of catalase) was prevented, an observation suggestive for a potential therapeutic role of angiotensin receptor blockers in pathologies associated with MAO overexpression [56].

The beneficial effects of MAO inhibition in the setting of hypertension were noticed also in spontaneously hypertensive rats (SHR). *Ex vivo* incubation of aortic rings (organ bath experiments) with both MAO-A and B inhibitors reversed the impaired vascular function by improving endothelium-dependent relaxation [57]. In the same experimental model, Poon et al. reported an increased protein expression of MAO-A in basilar arteries harvested from SHR as compared to the corresponding controls (WKY). Furthermore, an endothelium-denuded basilar artery (to eliminate the possible contribution of NO) was isolated for tension measurement and showed an exaggerated 5-HT-elicited contractile response that was reversed in the presence of the MAO-A inhibitor clorgyline or the ROS scavenger polyethylene glycol-catalase, respectively [58].

The beneficial role of MAO inhibition in humans was further confirmed in a subsequent study performed in patients subjected to coronary artery bypass and from whom mammary arteries were isolated for vasomotricity experiments. Indeed, a significant improvement of relaxation after MAO inhibition was recorded; of note, all patients included in this study were hypertensive [11]. Collectively, these data strongly suggest that MAO-related ROS production contributes to the development of hypertension and MAO inhibition is able to partially restore vascular function.

However, since MAO is the major enzyme responsible for catecholamine degradation, the well known “cheese-effect” associated to MAO inhibition in clinical settings has to be mentioned. In particular, after the ingestion of fermented cheese rich in tyramine (the norepinephrine precursor), treatment with MAO inhibitors can lead to vasoconstriction and hypertensive crisis [59]. Importantly, the effect was not observed after the administration of the novel reversible MAO inhibitors, such as moclobemide [60].

## 5. MAO and Diabetes Mellitus

With respect to the role of MAO in the development of oxidative stress-mediated endothelial dysfunction in diabetes, a rather limited number of studies are available in the literature. The upregulation of both MAO isoform expressions in aortas isolated from streptozotocin-induced diabetic rats was reported. Irreversible MAO inhibition with clorgyline for MAO-A and selegiline for MAO-B was able to both reduce the vascular contractility and improve the endothelium-dependent relaxation. Moreover, MAO inhibitors were able to reduce the level of hydrogen peroxide in diabetic aortic samples by approximately 50% [12]. Also, in Zucker diabetic fatty rats, an experimental model of type II diabetes, incubation of aortic rings with MAO inhibitors reduced the oxidative stress by more than 50% and improved vascular reactivity [61].

**Figure 3:**
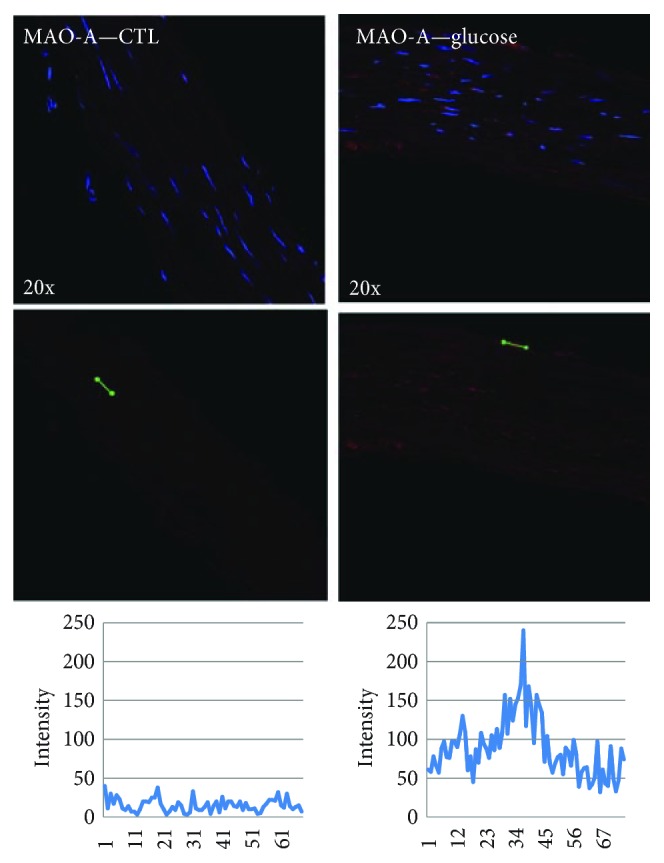
Glucose stimulation (400 mg/dl, 12 h) leads to increased MAO-A expression in rat aorta (immunofluorescence: blue—DAPI and red—MAO-A).

In order to investigate whether the upregulation of vascular MAO in diabetes is a direct consequence of hyperglycemia, we incubated rat aortic samples with high glucose (400 mg/dl, 12 h); a significant increase in MAO-A expression in immunohistology was observed (Figure 3). More recently, it was reported that ex vivo incubation of aortic rings harvested from diabetic rats with vitamin D was able to reduce MAO expression and improve vascular reactivity [62].

As for humans, it has been reported that MAO is expressed in mammary arteries harvested from patients with coronary heart disease and preserved ejection fraction subjected to revascularization therapy, regardless of the presence or absence of diabetes; of note, in these patients MAO-B was the predominant isoform. In diabetic (and nondiabetic) patients, MAO inhibitors were able to improve vascular relaxation and mitigate the oxidative stress [11]. In a recent study, Manni et al. also reported a significant increase in the activity and expression of both MAO isoforms (MAO-A/B) in ventricular samples from end-stage ischemic failing hearts (but not in the case of the nonischemic ones). Moreover, differences were found in the activities of the enzymes responsible for the metabolism of MAO's by-products, with a significant increase in both catalase and aldehyde dehydrogenase-2 in the failing left ventricle, whereas in the right one statistical significance was reached only for the latter [35]. The chamber differences in ROS regulation (including the inability to increase the antioxidant defense in conditions associated with MAO upregulation) may account for the reduced capacity of the right ventricle to compensate for cardiac stress such as pulmonary hypertension [63].

The increased oxidative stress in the cardiovascular system in the setting of diabetes mellitus has been systematically reported. The major pathways responsible for the overproduction of ROS are represented by the following: the increased formation of advanced glycation end-products [64], the polyol pathway [65], the activation of protein kinase C (PKC) [66], and the overactivity of the hexosamine pathway [67]. The cellular sources of ROS associated with these biochemical pathways have been classically represented by the respiratory chain, NADPH oxidases, and/or eNOS uncoupling.

In line with the abovementioned results, the role of MAO as a novel source of ROS in diabetes should be acknowledged. Also, these data suggest once more that MAO inhibitors might be useful in restoring endothelial response in clinical conditions associated with elevated oxidative stress and vascular dysfunction, such as coronary artery disease and diabetes.

## 6. MAO and Obesity/Metabolic Syndrome

Obesity is considered nowadays as one of the threatening pandemics of the 21st century, because its prevalence continues to rise worldwide (including among children and particularly in developing/low-income countries) and it is strongly associated with diabetes, cardiovascular disease, and chronic inflammation [68, 69]. Excessive amounts of adipose tissue (especially visceral) are responsible for the generation of high amounts of reactive oxygen species (ROS) that are linked with obesity-related pathologies [70]. Despite the unequivocal role of MAO as mitochondrial contributors to ROS production in the cardiovascular system in experimental and clinical settings, the potential role of these enzymes in obesity, one of the most frequent chronic metabolic disease associated with a state of increased oxidative stress, appears to be overlooked. A number of studies observed an increased MAO activity in obese mice [71], dogs [72], and pigs [73], but the translation of this observation in humans was not clarified so far.

In a previous study performed in patients with coronary heart disease and in patients with an indication for revascularization, the presence of MAO in the perivascular adipose tissue of the mammary arteries has been reported [11]. More recently, it has been observed that MAO-A inhibition corrects endothelial dysfunction in mesenteric artery branches isolated from obese patients; indeed, ex vivo acute incubation of the arterial rings with the MAO-A inhibitor, clorgyline, significantly improved the endothelium-dependent relaxation and decreased the level of H_2_O_2_ (Adrian Sturza, unpublished data).

Importantly, a recent study reported that the MAO-B inhibitor, selegiline, reduced subcutaneous and visceral adiposity as well as inflammation of white adipose tissue in a rat model of diet-induced obesity (with high-fat and high-sucrose diet); these effects strongly suggest a beneficial role of MAO inhibitors as an adjuvant therapy in patients with obesity, metabolic syndrome, and type 2 diabetes [74].

## 7. MAO and Chronic Kidney Disease

The contribution of MAO-related oxidative stress to the endothelial dysfunction associated to chronic kidney disease has also been reported. In this study, brachial artery collaterals were harvested from patients with end-stage renal disease (ESRD) with indication of hemodialysis during the surgical intervention for the arteriovenous (AV) fistula formation. Accordingly, MAO inhibition reduced the level of oxidative stress, improved the vascular reactivity by decreasing contractility, and increased relaxation in vascular samples from ESRD patients [75]. In line with this finding, MAO inhibitors might be useful for treating vascular dysfunction in the context of AV fistula maturation, the “lifeline” for hemodialysis patients.

## 8. Conclusion

Oxidative stress and inflammation are the most common pathomechanisms that lead to vascular remodeling and fibrosis in hypertension [76], micro- and macrovascular complications in diabetes [77], and progression of chronic kidney disease [78]. Accordingly, pharmacological strategies aimed at mitigating both processes, such as MAO inhibition with the new generation of reversible and selective drugs, will provide a rational therapeutic approach in all these various pathologies. In this respect, we provided translational evidence for the beneficial effects of MAO inhibitors in human samples. The challenge remains for the coming years to recapitulate these effects with the *in vivo* administration of these drugs in adequately powered clinical trials.
